# Automatic Context-Specific Subnetwork Discovery from Large Interaction Networks

**DOI:** 10.1371/journal.pone.0084227

**Published:** 2014-01-01

**Authors:** Ashis Saha, Aik Choon Tan, Jaewoo Kang

**Affiliations:** 1 Department of Computer Science and Engineering, Korea University, Seoul, Korea; 2 Department of Medicine/Medical Oncology, University of Colorado Anschutz Medical Campus, Aurora, Colorado, United States of America; 3 Interdisciplinary Graduate Program in Bioinformatics, Korea University, Seoul, Korea; Institute for Research in Biomedicine, Spain

## Abstract

Genes act in concert via specific networks to drive various biological processes, including progression of diseases such as cancer. Under different phenotypes, different subsets of the gene members of a network participate in a biological process. Single gene analyses are less effective in identifying such core gene members (subnetworks) within a gene set/network, as compared to gene set/network-based analyses. Hence, it is useful to identify a discriminative classifier by focusing on the subnetworks that correspond to different phenotypes. Here we present a novel algorithm to automatically discover the important subnetworks of closely interacting molecules to differentiate between two phenotypes (context) using gene expression profiles. We name it COSSY (COntext-Specific Subnetwork discoverY). It is a non-greedy algorithm and thus unlikely to have local optima problems. COSSY works for any interaction network regardless of the network topology. One added benefit of COSSY is that it can also be used as a highly accurate classification platform which can produce a set of interpretable features.

## Introduction

Biological systems are complex in nature; individual molecular components (e.g., genes and proteins) interact with each other in specific networks to exert their functions. The development of new technologies and powerful computational algorithms to sequence and characterize genomes have enabled researchers to acquire and analyze tens of thousands of ‘omic’ data points across the genetic and epigenetic changes within biological systems. One of the main difficulties in the gene expression analysis is the size of the ‘omic’ data. Due to the large number of genes, the complexity of the algorithms becomes very high.

The common practice is to rank the individual genes according to a similarity or dissimilarity score and then use the important genes for prediction. However, Risch (2000) suggested that a combination of genes is more apt for common disease classification than individual genes [1]. Marchini, Donnelly, and Cardon (2005) illustrated the effect that gene-gene interactions have on complex diseases [2]. Moreover, recent research suggests that molecular networks, rather than random sets of genes, can help find underlying physiological states associated with disease and understand complex biological processes [3]. Such approaches are more objective and robust in their ability to discover sets of coordinated differentially expressed genes among pathway members and their association to a specific biological phenotype. These analyses may provide new insights linking biological phenotypes to their underlying molecular mechanisms, as well as suggesting new hypotheses about the pathway membership and connectivity.

For this reason, researchers are now concentrating on gene-gene interactions instead of on individual gene analyses. Geman et al. (2004) introduced the relative expression reversal concept of top-scoring pair (TSP) as a classification approach for gene expression profiles [4]. They attempted to figure out the pairs of genes whose expression levels typically invert from one class to another. This concept has been improved by majority voting of the 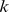 top scoring pairs (*k*-TSP) [5]. Chopra et al. (2010) also introduced their version of gene-pairing which they call *doublet* [6]. Instead of using only the inverse relations of the expression levels, they provided a framework for using different kinds of relations between two genes. Methods that extended the same concept for finding triplets [7] and other combinations of gene pairs [8], [9] have been developed.

Although these methods produce highly accurate results, they only consider the interaction of a few genes at a time which is still a small number to imply the biological process of a disease. Conversely, gene set enrichment analysis methods such as the Gene Set Enrichment Analysis (GSEA) [10] have improved the ability to identify candidate genes that are correlated with a disease state by exploiting the idea that gene expression alterations might be revealed at the level of biological pathways or co-regulated gene sets, rather than at the level of individual genes. However, the interactions (or topology) of the identified subset of genes within the predefined gene sets are not captured by these methods.

The availability of large-scale molecular interaction data such as those collected in KEGG (Kyoto Encyclopedia of Genes and Genomes) [11] and STRING (Search Tool for the Retrieval of Interacting Genes/Proteins) [12] provides an opportunity to incorporate these interactions into omic data analysis. The advantage of these network-based approaches is that they can fully describe the complex biological processes driving disease phenotypes. Most network-based approaches attempted to identify informative subnetworks - a set of genes that interact directly and have differential expression patterns - for disease classification. These approaches attempted to find these subnetworks using a greedy approach, starting with a small network (typically a single node) and adding nodes greedily until the score is improved [13]–[16]. However, they may suffer from local-optima problems and may not even work for a sparse network such as KEGG because their greedy expansion process could halt imgmaturely due to sparse connectivity. Alternatively, some approaches, such as DIRAC (Differential Rank Conservation)[17], utilized user-defined and pre-partitioned subnetworks for classification.

Here we propose a novel COntext-Specific Subnetwork discoverY (COSSY) algorithm to automatically discover the important subnetworks to differentiate between two phenotypes (context). COSSY finds the important subnetworks using a non-greedy approach. Unlike the greedy approach, our approach first divides the network, and then ranks the subnetworks based on the biological context of interest. We demonstrate that COSSY works for any network regardless of its topology. We show that COSSY is capable of identifying discriminative subnetworks while achieving classification results comparable to those of other state-of-the-art methods. Finally, we illustrate that the subnetworks identified by COSSY provide meaningful biological insights from high-throughput datasets.

### COSSY Algorithm

Let 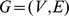 be a molecular interaction network (graph) where each vertex in 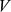 denotes a molecule (or a set of molecules) and each edge in 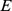 denotes an interaction between molecules. A molecule can be a gene, a protein, a nucleic acid or a similar biochemical object. Let 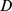 be the training dataset containing the microarray expression profiles of 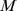 samples, where each profile consists of 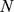 probes. 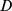 can be represented as an 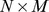 matrix where 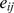 denotes the expression value of the 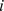-th probe, 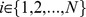, in the 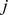-th sample, 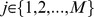. The row vector 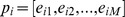 thus represents the 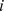-th probe across 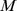 samples and the column vector 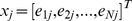 represents the 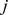-th sample across 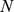 probes. Let 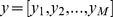 be the vector of the class labels (context) for 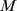 samples where 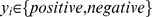. Our task is to find the subnetworks (subgraphs) of 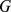 to predict the class (context) of a new sample 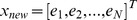.

**Figure 1:**
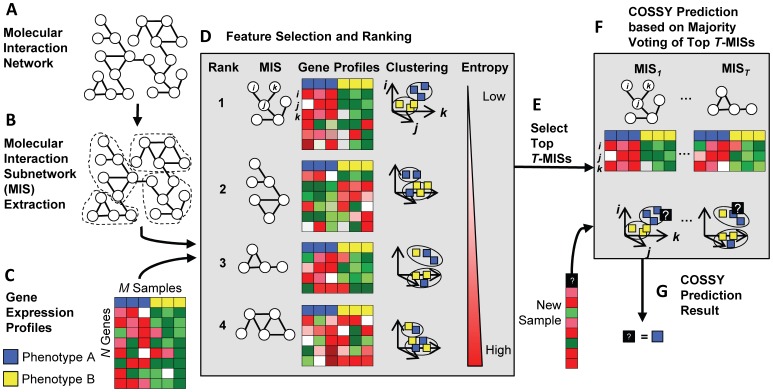
Overview of COSSY. Communities are extracted from the molecular interaction network to generate Molecular Interaction Subnetworks (MISs)(A–B). Each MIS is mapped to microarray probes and all the samples are clustered according to the expression pattern of a certain number of highly differentially expressed probes (3 probes in this example figure). MISs are ranked by the entropy score which is lowest when every cluster contains only one type (phenotype) of samples (C–D). Finally, the top 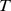 MISs cast votes to predict the context (phenotype) of a new sample. The voting depends on the proportion of different types of samples in the cluster closest to the new sample (E–G).

To give an overview, we first partition the molecular interaction network 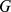 to generate the molecular interaction subnetworks. Each subnetwork is then ranked using a clustering approach and finally the top 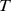 subnetworks vote to predict the context of the new sample, 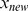 (Figure 1).

### Molecular Interaction Subnetwork (MIS)

**Figure 2:**
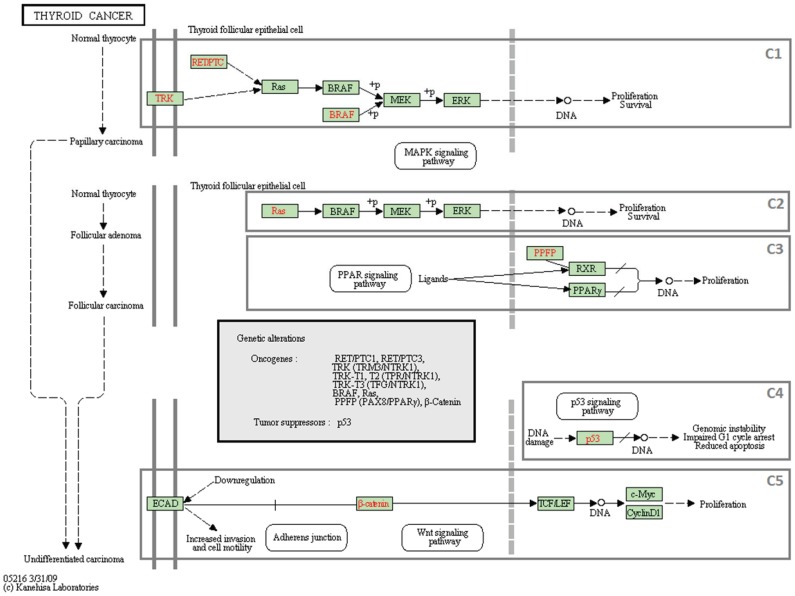
The thyroid cancer pathway in KEGG (ID: hsa05216). The top rectangle marked by C1 shows that REDPTC and TRK have an indirect effect on the activation of Ras. Ras then activates BRAF, and BRAF phosphorylates MEK which in turn phosphorylates ERK. The result of this path is proliferation survival. This pathway has five connected components (C1–C5). Among them, C2 is actually a subset of C1, and the others are fully disconnected, i.e., there is no significant interaction between any pair of the components.

We use a biological knowledge-base in the form of a network in order to get biologically interpretable results. An important property of such a network is that it shows how a group of molecules interact with each other, often performing a molecular function. However, it is not necessary for all the molecules in a network to interact with each other. For example, some of the genes in the thyroid cancer pathway in KEGG (id: hsa05216) are not connected (Figure 2). Traditional pathway analyses consider all the molecules (genes) in the same pathway together. We believe that the non-interacting set of molecules should be considered separately. Therefore, we propose to include only the interacting molecules in a candidate set, which we call the *Molecular Interaction Subnetwork (MIS)*.

We further adjust the idea of an MIS for large number vertices in a connected network. Some pathways such as the cell cycle pathway in KEGG (id: hsa04110) contain a large number of genes all of which are connected to each other, although different parts of the pathway are annotated to have different functions. We believe that, it would not be wise to assume that each and every molecule in a large network responsible for a process would also be responsible for another process. Instead, we reasonably assume that a subset of molecules in one process may also affect the development of another process. Hence, we propose to divide the large network into pieces so that only the closely interacting molecules are in the same subnetwork. An MIS will contain all the molecules in such a subnetwork.

We utilize the community structure of a network –groups of nodes within which the edge densities are high, but between which they are low – to find the closely interacting subnetworks. We first build a community dendrogram as described by [4]. Smaller communities can be made by splitting the dendrogram from the top. Modularity, a measure of the quality of a particular division of a network, is based on the ratio of the number of edges within groups to the total number of edges in the network. The division improves as the modularity increases. Among all the available ones, the pair of groups of nodes whose integration would produce the highest modularity are joined until one group remains. This process can be presented as a dendrogram whose division from the top to the bottom would create smaller communities. If the community size is within an *appropriate range [minRange, maxRange]*, we consider it as an *appropriate community*. If the size is above the appropriate range, we cut the community dendrogram from the top. So we get two small communities and we recursively do this until the size is within the appropriate range. The partition process discards any branch whose size goes below the appropriate range and once the partition process is over, each node in the discarded branch is individually merged with the closest appropriate community. This merging step may cause some communities to grow above the appropriate range. For each of these communities, we create a new network taking all the nodes of the community and the edges among them from the original network, and then we apply the whole process to the new network. Algorithm 1, along with Algorithm 2–4, describes the MIS generation process in detail. The algorithm is illustrated in Figure S1 in Supporting Document S1.

**Algorithm 1 Generate MIS from network**

**Input:** 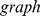, a molecular interaction network

**Output:** List of MISs generated from 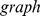

1: **for all** Connected component 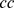 in 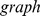 **do**

2: **if** 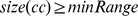 **then**

3: Generate MISs from 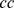 using Algorithm 2 and save them.

4: **eles**

5: Generate an MIS taking all the molecules in 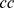 and save it

6: **end if**

7: **end for**

8: **return** all saved MISs

**Algorithm 2 Generate MIS from connected network**

**Input:** **Input:** 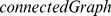, a connected network with at least 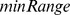 nodes.

**Output:** MISs generated from 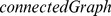.

1: 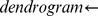 Build a community dendrogram as described by [18].

2: 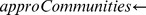 appropriate communities generated from 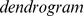 using Algorithm 3.

3: 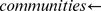 communities after merging the discarded nodes in step 2 with the closest appropriate communities using Algorithm 4.

4: **for all** community 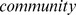 in 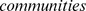 **do.**

5: **if** size(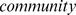)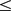 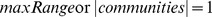 **then.**

6: Create and save an MIS taking all the molecules in 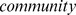.

7: **else.**

8: 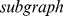 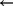 a subgraph of 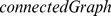 containing all the leaf nodes of 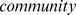 and all the edges among them in 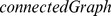.

9: Recursively generate and save MISs from 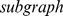.

10: **end if.**

11: **end for.**

12: **return** all saved MISs.

**Algorithm 3 Extract appropriate communities from dendrogram**

**Input:** 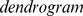, the community dendrogram.

**Output:** Appropriate communities extracted from 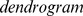.

1: 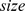 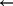 No. of leaf nodes in 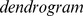.

2: **if** 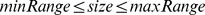 **then.**

3: Create a community of all the leaf nodes in 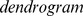 and save it.

4: **even if** 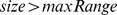 **then.**

5: Divide 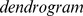 into 2 parts from the top - 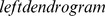 and 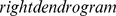.

6: Recursively extract and save appropriate communities from 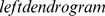 and 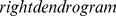.

7: **eles.**

8: Discard the 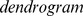.

9: **end if.**

10: **return** saved appropriate communities.

**Algorithm 4 Merge discarded nodes with given communities**

**Input:** 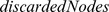, nodes not taken in any community; 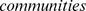, given communities.

**Output:** Communities with 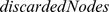 merged.

1: **repeat.**

2: 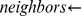 The subset of 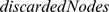 directly connected (1-hop distance) to some node in 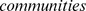 from the original network.

3: **for all** node 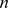 in 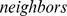 **do.**

4: 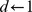.

5: 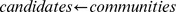.

6: **loop.**

7: 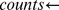 Count no. of 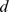-hop distant nodes (from 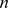) in each community in 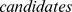. 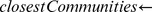 communities having 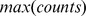 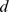-hop distant nodes from 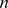.

9: **if** 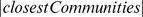  = 0 **then.**

10: Mark 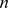
's membership to one community in 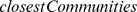 randomly.

11: **break** loop.

12:** else if** 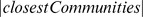  = 1 **then.**

13: Mark 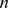
's membership to the only community in 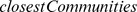.

14: **break** loop.

15:** else.**

16: 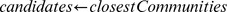.

17: 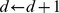.

18: **end if.**

19: **end loop.**

20: **end for.**

21: 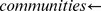 communities after merging every node in 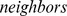 according to its membership.

22: 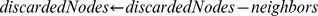.

23: **until** 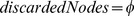.

24: **return** 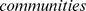.

### Feature Selection

It is necessary to map the molecules to the probes used in 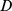. If one molecule is mapped to multiple probes we include all of them separately following the suggestion to consider each probe independently [19]. An MIS is selected if its molecules are mapped to at least a certain number of unique probes. Thus we avoid the unreliable effect of a single or low number of probes. For every selected MIS, we propose to take the same number of probes to consider them equally in the next step. In this study, we choose the five most differentially expressed probes in each MIS which we call the *representative probeset* for the MIS. We estimate the differential expression of a probe using a modified version of Welch’s t-statistic score (Section S1 in Document S1).

As one molecule can be mapped to multiple probes and can be in multiple nodes of some molecular interaction networks, the representative probesets of MISs may overlap with each other. We measure the overlap as the ratio of the number of common probes in the representative probesets of the two MISs over the number of probes in each representative probeset, 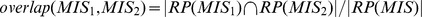, where RP(.) denotes the representative probeset of an MIS. We take two MISs with the highest overlap and combine them into one MIS by taking a union of all the molecules in both MISs. We continue to combine MISs with the highest overlaps as long as they are significant (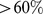 in this study).

### Ranking

**Figure 3:**
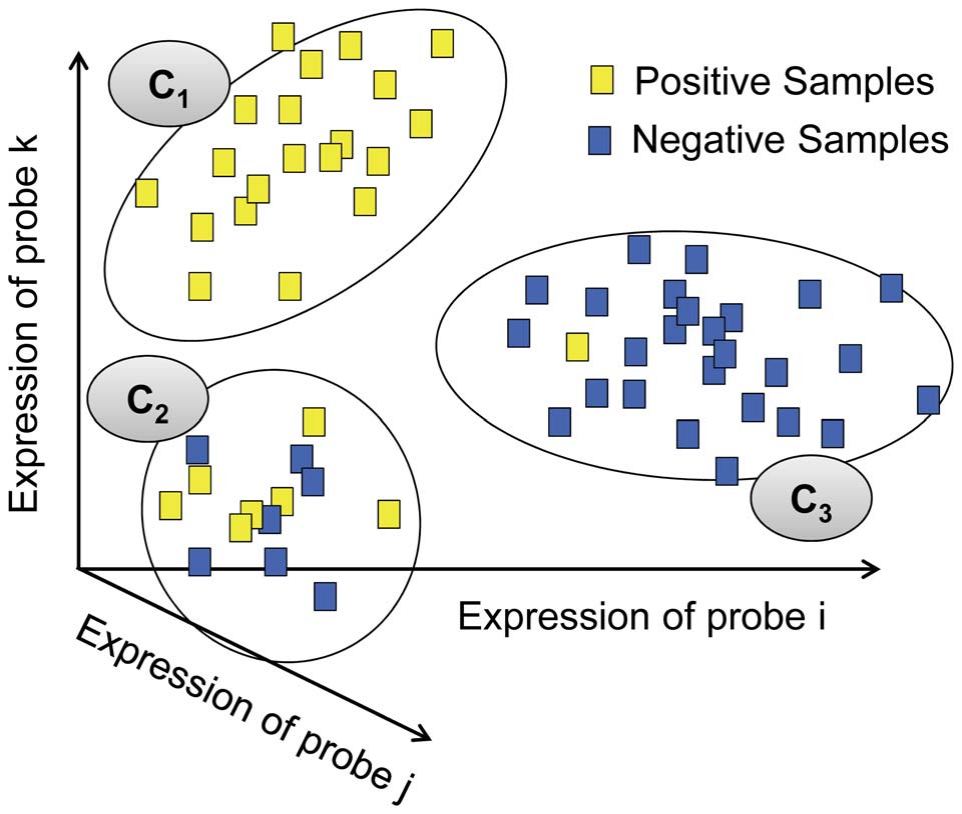
Ranking of MIS. Let three probes (i, j, and k) constitute the representative probeset of an MIS. We plot all the samples with the expression values of these probes in separate dimensions, and then we cluster the samples. If the samples in a cluster are mostly of one kind (such as 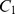 or 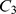), we can say that the cluster’s expression pattern represents the corresponding class (positive or negative). The ranking of an MIS producing such clusters should be high.

We rank the MISs based on the expression pattern of their representative probesets. We cluster all the samples using the expression values of the representative probeset. Each cluster represents a separate pattern. If a specific pattern corresponds to a class, we can expect that almost all the samples in the corresponding cluster will be of the same class (Figure 3). Based on this behavior, we propose an entropy-based score to rank the MISs.

Let us consider that all the samples in dataset 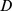 have been divided into 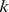 clusters 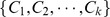 using the expression values of all the probes in an MIS representative probeset. Each cluster 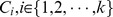 contains 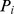 positive samples and 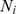 negative samples. We normalize the number of positive and negative samples in each cluster by dividing it by the total number of positive and negative samples in the dataset, respectively. The normalized number of positive samples is 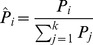 and the normalized number of negative samples is 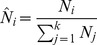. The entropy of cluster 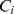 is then defined as
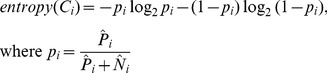
(1)


The entropy of the MIS is then defined as
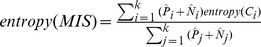
(2)


The range of each cluster’s entropy is 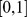. Consequently, the entropy of an MIS is always within 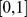. If all the samples in every cluster are of one kind (same class), then the entropy becomes zero. In contrast, it becomes one if every cluster contains an equal proportion of positive and negative samples. MISs are sorted by their entropies in an increasing order, i.e., the MISs with the lowest and highest entropies are at the top and bottom of the ranking, respectively.

### Context Prediction

The context (or class) of a new sample 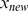 is predicted from the weighted voting by the top 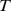 MISs 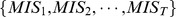. 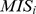 votes for the positive and negative class with the weight of 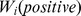 and 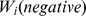, respectively (Eqs. 3 and 4).
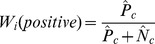
(3)

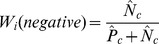
(4)where 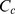 is the closest cluster to 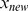 among the clusters produced by 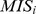, and 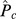 and 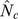 are the normalized numbers of the positive and negative samples, respectively, in 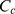. The sums of the voting weights by the top 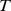 MISs for the positive class and the negative class are 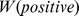 and 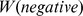, respectively (Eqs. 5 and 6).



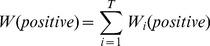
(5)

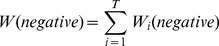
(6)


The class, with a voting weight that is higher than the other, is predicted to be the class of the new sample, 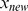. However, if both weights, 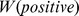 and 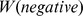, become equal, the class is predicted from *binary voting*: a class gets the full weight (i.e., one) if its number of samples surpasses that of the other class in a nearby cluster; otherwise it gets zero (Section S2 in Supporting Document S1). Binary voting would take place infrequently as we use only the top ranked MISs. In fact, it did not occur in any of our experiments. The binary voting system guarantees the winner when 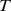 is odd. The classification rule is finally expressed as below (Eq. 7).
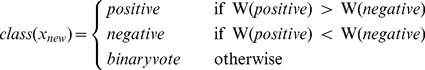
(7)


## Datasets, Implementation and Evaluation Method

### Microarray Datasets

We tested COSSY on seven publicly available cancer datasets, as shown in Table 1. In this paper, we refer to the datasets by the names mentioned in the first column of Table 1. The purpose of the Leukemia microarray dataset is to identify genes that distinguish Acute Myeloid Leukemia (AML), a deathly disease, from Acute Lymphoblastic Leukemia (ALL) [20]. It contains expression levels of 7129 probes from 72 samples (ALL:47, AML:25). We used dataset *B* from [21], which contains 25 classic and 9 demoplastic medulloblastoma samples, as the CNS dataset. The samples of the DLBCL dataset are divided into 2 categories: Diffuse Large B-Cell Lymphoma (DLBCL) and Follicular Lymphoma (FL) [22]. The Prostate1 dataset, distinguishing between prostate tumors and non-tumor prostates (normal), used 102 high-quality expression profiles from [23]. The Prostate3 dataset includes 24 prostate tumor and 9 normal samples from [24]. The goal of the Lung dataset is to differentiate between Malignant Pleural Mesothelioma (MPM) and Adenocarcinoma (ADCA) of the lung [25]. Finally, the *GCM_total* dataset from [26], which contains 90 normal and 190 tumor samples of several types of cancer, constitutes the GCM dataset. The sources of these datasets have been listed in Table S1 in Supporting Document S1.

**Table 1:** Microarray Datasets.

Dataset Name	#Probes	Positive Class (#samples)	Negative Class (#samples)	Reference
Leukemia	7129	AML (25)	ALL (47)	[20]
CNS	7129	Demoplastic (9)	Classic (25)	[21]
DLBCL	7129	DLBCL (58)	FL (19)	[22]
Prostate1	12600	Tumor (52)	Normal (50)	[23]
Prostate3	12626	Tumor (24)	Normal (9)	[24]
Lung	12533	MPM (31)	ADCA (150)	[25]
GCM	16063	Tumor (190)	Normal (90)	[26]

The first column, ‘Dataset Name’, indicates the name of the microarray dataset used in the manuscript. ‘#Probes’ shows the number of probes present in the dataset. The third and fourth columns contain the name of the positive and negative class, respectively, followed by the number of samples of that class. The last column shows the reference the dataset was collected from.

### Implementation of COSSY

COSSY is implemented as an R package. It requires R (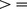
2.15.3) along with utils, stats, limma, and igraph (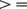
0.6.5) packages. COSSY has been tested on Windows, Linux, and Mac operating systems. The package and data used in this study are available for download on our website: http://infos.korea.ac.kr/cossy/.

### Experimental Settings

We first performed the quantile normalization over the training dataset and then standardized the probe data using z-score, 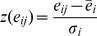, where 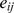 is the expression value of the 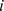-th probe in the 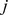-th sample; 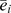 and 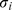 are the mean and standard deviation of the expression values, respectively, of the 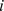-th probe. Ward’s minimum variance agglomerative hierarchical clustering [27] was used with euclidean distance. The dendrogram produced by Ward’s method was cut into 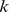 groups to form 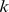 clusters. The number of clusters, 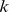, was chosen from the *rule of thumb*, 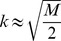, where 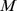 is the number of samples [28]. To avoid the effect of noisy data, the median of the data points in each cluster was taken as its centroid. A new data point would fall in the cluster whose centroid is the closest to the new point. Finally, 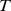, the number of the top MISs to vote, was selected experimentally. We experimented with 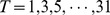 and used the minimum 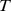 that produced the highest accuracy.

### Cross Validation

We validate the classification of COSSY by standard *Leave-One-Out Cross-Validation (LOOCV)*. For each sample 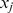 in 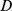, we train the classifier using the remaining (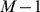) samples and then the classifier predicts the class of 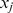. The LOOCV accuracy is the fraction of all the samples that are correctly classified.

## Results

### Networks Generated from KEGG and STRING

We applied COSSY on two well-known molecular interaction networks: KEGG and STRING. We chose KEGG and STRING, as they are both very different in nature. KEGG is composed of a number of disjoint pathways where the same molecule can be present in multiple pathways. In contrast, a molecule in STRING can have only one node. The nodes in KEGG may represent different types of objects (gene, gene product, chemical compound, etc.) while the nodes in STRING represent proteins. A KEGG node may have multiple molecules, while a STRING node always represents only one protein. The topologies of these two networks are also significantly different. STRING shows a much higher clustering tendency than KEGG. In this study, we used the unweighted and undirected graph of the *protein networks* – the network of the genes and their interactions – from the human signaling pathways in KEGG (release 62.0), and the human protein-protein interactions – both experimental and predicted with high confidence (*score*>*0.7*) – in STRING (release 9.05). A few properties of the networks are shown in Table 2.

**Table 2:** Network Properties of KEGG and STRING.

Network	TotalNodes	Gene (Protein) Nodes	Total Edges	ConnectedComp.	Avg. Node Degree	Max Node Degree	ClusteringCoefficient
KEGG	19568	10691	10728	4494	1.84	43	0.19
STRING	14250	14250	215800	182	30.30	1110	0.61

The ‘Total Nodes’ column contains the total number of nodes available in the network while the ‘Gene (Protein) Nodes’ column shows the number of nodes with at least one gene in KEGG (or one protein in STIRING). The fourth and fifth columns contain the total number of edges, and the number of connected components having at least one gene (or protein), respectively. ‘Avg. Node Degree’ represents the number of edges a node has on average. ‘Max Node Degree’ denotes the maximum number of edges a node has in the network. ‘Clustering Coefficient’ is the ratio of the triangles to the connected triples in a graph.

As described before, we try to keep the size of the MISs within the *appropriate range*. We experimented with different ranges (Table S2 and S3 in Supporting Document S1) and chose the range with the highest accuracy: 5–15 for KEGG and 5–25 for STRING. Here we should mention that the sizes of some MISs may be below or above the range (Table 3). We did not discard the smaller MISs in the MIS generation step, as their number of probes may be sufficient. Also, the bigger MISs could not be further divided because of their special topologies, e.g., a star network or a highly dense network (Figure S2 in Supporting Document S1). We used bioDBnet [29] to map the genes or proteins to the microarray probes.

**Table 3:** Molecular Interaction Subnetwork Size.

Network	Appropriate Range	MIS below the range	MIS within the range	MIS above the range
KEGG	5–15	1925	629	9
STRING	5–25	170	847	23

The table shows the number of MISs with a total of nodes below, within, and above the appropriate range.

### Features Selected from Each Dataset

**Figure 4:**
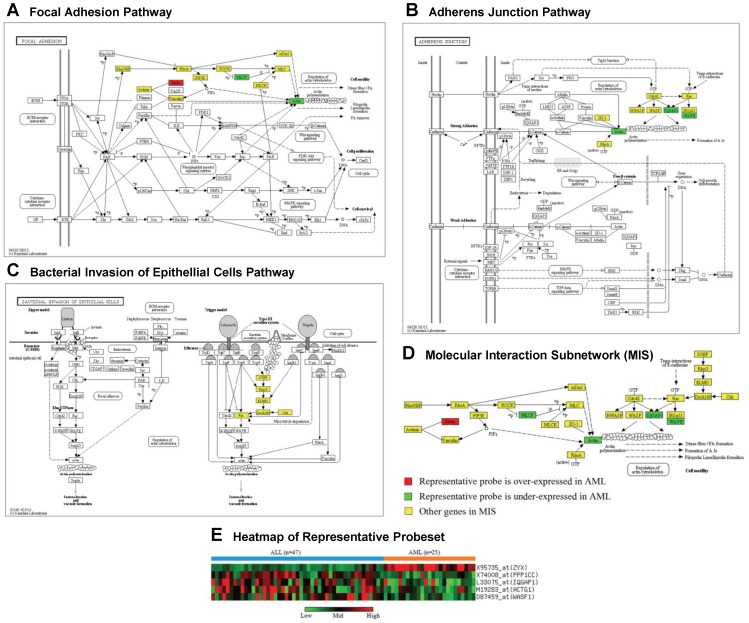
The top ranked KEGG MIS in the Leukemia dataset. A–C) Three overlapped subnetworks from three different pathways constitute the MIS. D) The merged MIS is shown here. E) The expression heatmap of the representative probeset of the MIS is shown here.

The representative probesets selected from each dataset using KEGG and STRING are shown in Table S4 and S5 in Supporting Document S1, respectively. Figure 4 demonstrates the top ranked KEGG MIS in the Leukemia dataset containing two types of samples: Acute Myeloid Leukemia (AML), and Acute Lymphoblastic Leukemia (ALL). The MIS was taken from three overlapped subnetworks from three different pathways: 1) Focal Adhesion Pathway (id: hsa04510), 2) Adherens Junction Pathway (id: hsa04520), and 3) Bacterial Invasion of Epithelial Cells Pathway (id: hsa05100). One probe (affymetrix id: X95735_at, gene symbol: ZYX) in its representative probeset is over-expressed in AML, while the others are under-expressed.

### Classification Accuracy

**Figure 5:**
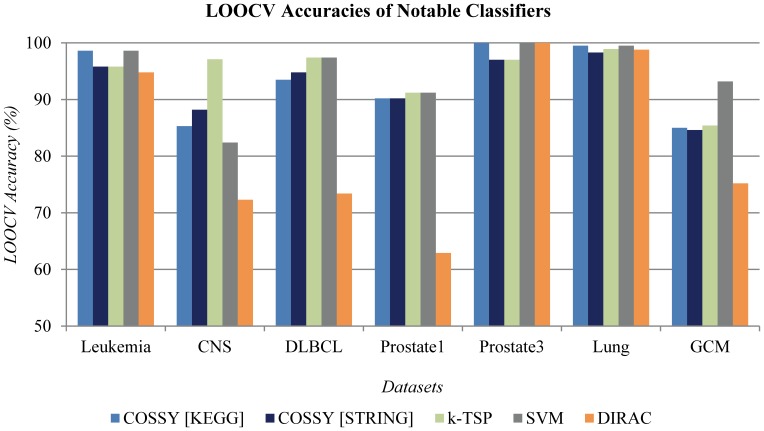
LOOCV accuracy of five notable classifiers. COSSY [KEGG] and COSSY [STRING] stand for COSSY using KEGG and STRING, respectively. *k*-TSP and DIRAC are the classification algorithms described in [5] and [17], respectively. SVM stands for the Support Vector Machine algorithm.

The LOOCV accuracies on the seven cancer datasets are shown in Table 4. COSSY achieved LOOCV accuracies of 93.2% and 92.7% using KEGG and STRING, respectively, which is comparable to the state-of-the-art classifiers, *k*-TSP and SVM (Support Vector Machine), and better than other classifiers (DIRAC, TSP, Doublet, PAM, Decision Tree, Nearest Neighbor, Nave Bayes) including a recent network-based approach, DIRAC [7]. Figure 5 shows the classification accuracies of five classification methods: COSSY using KEGG, COSSY using STRING, *k*-TSP, SVM, and DIRAC. From this figure, it is evident that COSSY’s performance on the five datasets (Leukemia, DLBCL, Prostate1, Prostate3, and Lung) is almost equal to that of *k*-TSP and SVM; *k*-TSP outperforms others using CNS, and SVM outperforms others using GCM significantly. Notably, COSSY’s performance on six datasets is significantly better than that of DIRAC; COSSY’s performance on the remaining dataset (Prostate3) is equal to that of DIRAC. On average, COSSY’s accuracy is more than 10% higher than that of DIRAC (COSSY using KEGG - 93.2%, COSSY using STRING - 92.7%, DIRAC - 82.5%). We also report the Area Under Curve (AUC) values of Receiver Operating Characteristic (ROC) curves achieved in the cross validation by COSSY and DIRAC in Table 5. COSSY outperforms DIRAC in terms of AUC (COSSY using KEGG - 0.948, COSSY using STRING - 0.945, DIRAC - 0.813). Even though a random classifier, which predicts the majority class, may have high accuracies due to the imbalance in the number of positive and negative samples in the datasets, the random classifier is unlikely to achieve as high AUCs as COSSY achieved.

**Table 4:** LOOCV accuracy (%) of classifiers.

Method	Leukemia	CNS	DLBCL	Prostate1	Prostate3	Lung	GCM	Average
COSSY [KEGG]	98.6	85.3	93.5	90.2	100.0	99.5	85.0	93.2
COSSY [STRING]	95.8	88.2	94.8	90.2	97.0	98.3	84.6	92.7
DIRAC	94.8	72.3	73.4	62.9	100.0	98.8	75.2	82.5
*k*-TSP*	95.8	97.1	97.4	91.2	97.0	98.9	85.4	94.7
TSP*	93.8	77.9	98.1	95.1	97.0	98.3	75.4	90.8
SVM*	98.6	82.4	97.4	91.2	100.0	99.5	93.2	94.6
Doublet [Sign-DT]+	93.1	82.4	97.4	86.3	97.0	98.3	85.0	91.3
Doublet [Sumdiff-DT]+	91.7	70.6	97.4	82.4	87.9	95.0	81.4	86.6
Doublet [Mul-DT]+	84.7	55.9	97.4	86.3	90.9	92.3	83.2	84.4
Decision Tree (DT)*	73.6	67.7	80.5	87.3	84.9	96.1	77.9	81.1
Nave Bayes*	100.0	82.4	80.5	62.8	90.9	97.8	84.3	85.5
*k* Nearest Neighbor*	84.7	76.5	84.4	76.5	87.9	98.3	82.9	84.5
PAM*	97.2	82.4	85.7	91.2	100.0	99.5	79.3	90.7

The leftmost column contains the names of the methods; the rightmost column shows the average accuracy of each method for seven datasets, and other columns show the accuracy (%) for individual datasets. ‘COSSY [KEGG]’ and ‘COSSY [STRING]’ represent COSSY using KEGG and STRING, respectively. ‘DIRAC’ is the algorithm proposed in [17] whose LOOCV accuracies have been calculated using the matlab code published with the paper. *k*-TSP and TSP denote the classification algorithms described in [5] and [4], respectively. SVM stands for Support Vector Machine. ‘Doublet [Sign-DT]’, ‘Doublet [Sumdiff-DT]’, and ‘Doublet [Mul-DT]’ denote the classification methods using Sign-Doublet, Sumdiff-Doublet, and Mul-Doublet, respectively, with decision trees as described in [6]. The last three rows contain the loocv accuracies using Nave Bayes, *k* Nearest Neighbor, and PAM classifier, respectively.

Results obtained from [5].

Results obtained from [6].

**Table 5:** Area Under Curve (AUC) values of COSSY and DIRAC for different datasets.

Dataset Name	AUC of COSSY using KEGG	AUC of COSSY using STRING	AUC of DIRAC
Leukemia	**0.986**	0.985	0.948
CNS	0.862	**0.876**	0.726
DLBCL	**0.985**	0.976	0.636
Prostate1	0.909	**0.918**	0.635
Prostate3	**1.000**	0.972	**1.000**
Lung	**0.999**	**0.999**	0.990
GCM	**0.896**	0.889	0.757
Average	**0.948**	0.945	0.813

AUC has been calculated using the ROCR package in R [35]. The best AUC for each dataset is highlighted in bold face.

### Interpretation of the COSSY Results on the Leukemia Dataset

As we discussed in this paper, the advantage of COSSY is its ability to automatically discover the important subnetworks to differentiate between two phenotypes (context-specific). We used the Leukemia dataset to illustrate this interpretation of the subnetworks identified by COSSY. As demonstrated in the results section, we selected the top five differentially expressed genes from each subnetwork for classification. Not surprisingly, the top subnetwork identified by COSSY contains ZYX (zyxin), which has been previously identified as the top differentially expressed gene to distinguish AML from ALL in this dataset [20]. Other genes previously identified as gene signatures to distinguish AML from ALL include CTSD, LYN, MYB [20].

**Figure 6:**
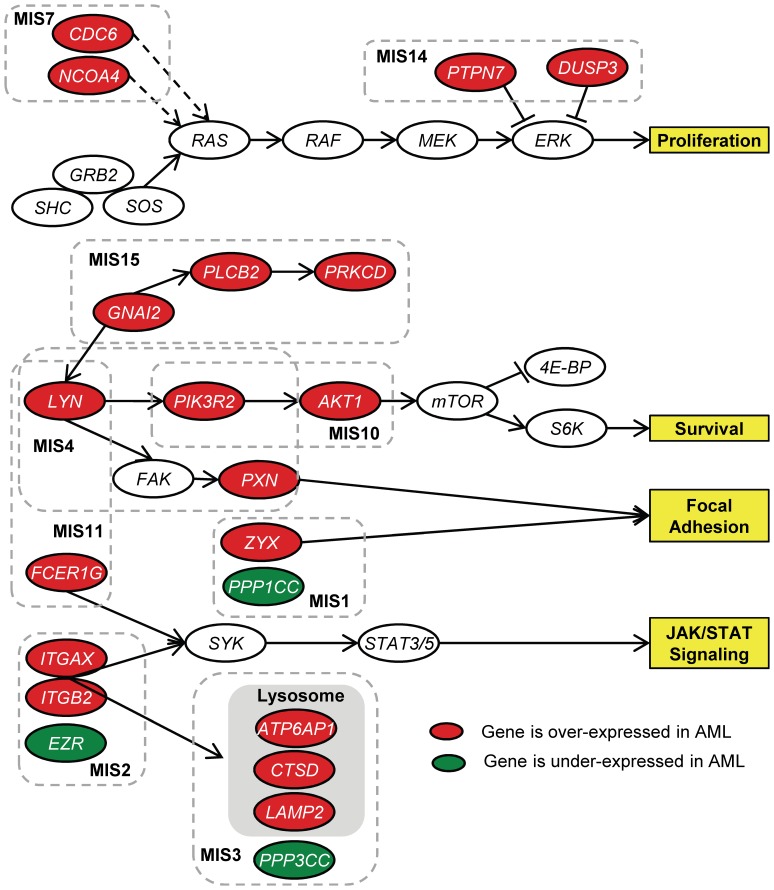
Stitching of the MISs found from the Leukemia dataset. The number at the end of the name of an MIS indicates its rank.

Examining the over-expressed genes in AML across the top 15 subnetworks (Table S4 in Supporting Document S1), COSSY identified LYN/PI3K/AKT signaling (LYN, PIK3R2, AKT1), lysosome complex (LAMP2, CTSD, ATP6AP1), and integrin signaling pathways (ITGAX, ITGB2, FCER1G) as key pathways in driving this disease. By querying the core genes identified from the top MISs to the KEGG pathways, we could ‘stitch’ these subnetworks (MISs) together as illustrated in Figure 6. This ‘stitched’ network revealed three key pathways that have been described to be deregulated in AML. In particular, LYN, an Src-family kinase member, has been found to be over-expressed in AML [20], [30]. Previous studies described LYN to be linked to mTOR [30]; we revealed this link between LYN and mTOR via PIK3R2 and AKT1 by stitching these subnetworks together in this analysis.

PI3K/AKT signaling has been implicated as playing a critical role in AML [30]–[32]; also, inhibiting multiple components of this pathway might provide the therapeutic interventions to this lethal disease. From the stitched network, we identified ITGAX and ITGB2 as over-expressed in AML; these two integrin receptors interact with each other as well as with FCER1G. Recently, it has been found that the interactions of ITGAX/ITGB2 and FCER1G could signal SYK-dependent activation of the JAK/STAT pathway in AML, and this signaling axis might serve as a novel therapeutic target for AML [33].

Finally, we observed that the multiple components of the lysosome (CTSD, LAMP2, and ATP6AP1) were over-expressed in AML. This is an interesting finding as a recent paper demonstrated that using mefloquine, an anti-malaria drug, could disrupt lysosome in AML and increase cancer cell killing effects [34]. Furthermore, AML cells were less viable due to gene knock-down on LAMP2. This demonstrates that lysosome could be a new therapeutic target in AML [34]. From this example, we highlight that COSSY can identify interesting and context-specific subnetworks from microarray gene expression datasets. By stitching the core genes together from these subnetworks, novel biology could be discovered. This represents an added value to COSSY as compared to static pathway-based analyses such as Gene Set Enrichment Analysis.

## Discussion

### Gene Interaction

Conventional differential expression analyses focus on single gene markers. Even the gene pairs identified by *k*-TSP or *doublets* do not necessarily imply a biochemical interaction between the genes. COSSY, in contrast, takes biological interactions under consideration ensuring a differential expression pattern of the representative probeset instead of a differential expression of a single gene. Consequently, it provides an opportunity to discover the development process of a phenotype.

### Network Partition

Unlike GSEA and DIRAC, COSSY does not require any pre-partitioned subnetworks and is not limited by the quality of the predefined gene sets. COSSY can automatically generate the subnetworks. Importantly, these subnetworks may also be used with other types of analyses requiring pre-partitioned subnetworks, which is not yet possible from any of the existing subnetwork discovery methods.

### Non-greedy Approach

The existing subnetwork discovery methods [13]–[16] usually build subnetworks using a greedy approach that starts with a small network (typically a single node) and then adds more nodes greedily until the addition improves the quality. However, it may have local-optima problems. On the other hand, as our method follows the non-greedy approach – divides the network first and then calculates its effectiveness – local-optima problems are unlikely to occur. Moreover, the applicability of the previous greedy methods often depends on the network topology, which is explained in the following section.

### COSSY is Independent of Network Topology

Current subnetwork discovery methods are highly dependent on the network topology. The method by Chuang et al. (2007) [13] is likely to stop at a locally optimum node frequently where strict linear paths dominate in a network such as KEGG. Chowdhury et al. (2010) [14] use the set-cover-based algorithm where either all the positive or negative samples have to be covered. If the network contains small components, then it might not be able to cover the positive or negative samples completely. Dao et al. (2010) [15] utilize density-constrained clustering which would not work well on sparse networks such as KEGG. Su et al. (2010) [16] look for the discriminative linear paths and then combine them to build the subnetwork. In a sparse network, the linear paths may not intersect each other. In contrast, we showed that COSSY works well on networks with highly different topologies such as KEGG and STRING. The average LOOCV accuracies of COSSY are 93.2% and 92.7% when used with KEGG and STRING, respectively, and are high and close to each other.

### Subnetworks with Heterogeneous Characteristics

Chuang et al. (2007) [13] take the sum of the expressions of all the nodes in a subnetwork to aggregate its expressions, and thus the process favors the subnetworks with homogeneous characteristics where all the nodes are either positively or negatively expressed. The NetCover algorithm [14] also aggregates the expressions in the same way and suffers from the same problem. Instead of aggregating the expressions, COSSY uses clustering and thus it is capable of utilizing the heterogeneous nature to find the subnetworks that have both positively and negatively expressed nodes. For example, the representative probeset of the Leukemia MIS in Figure 4 contains one positively and four negatively expressed probes.

## Conclusion

In this paper, we have introduced a non-greedy algorithm, COSSY, to partition a molecular interaction network and find the important subnetworks discriminating between two phenotypes. COSSY can find subnetworks with heterogeneous characteristics in any network irrespective of the topology. Its accuracy is comparable to the state-of-the-art classifiers. We also illustrated an interpretation of the results to discover the development process of a disease.

## Supporting Information

Document S1 **Supporting information document.** This document contains seven sections (S1. Estimated t-score, S2. Binary Vote, S3. Dataset Download Sources, S4. An Experiment with the appropriate range, S5. Algorithm Illustration, S6. Irregular Network Topology, and S7. Representative probesets of the top MISs), two figures (S1. An illustration of the MIS generation, and S2. Topology of out-of-size MISs), and five Tables (S1. Dataset Download Sources, S2. LOOCV accuracy with different appropriate ranges from KEGG, S3. LOOCV accuracy with different appropriate ranges from STRING, S4. Representative probesets of the top MISs from KEGG, and S5. Representative probesets of the top MISs from STRING).
(PDF)

## References

[1] Risch NJ (2000) Searching for genetic determinants in the new millennium. Nature 405: 847–856. 10866211 10.1038/35015718

[2] Marchini J, Donnelly P, Cardon LR (2005) Genome-wide strategies for detecting multiple loci that influence complex diseases. Nature Genetics 37: 413–417. 15793588 10.1038/ng1537

[3] Schadt EE (2009) Molecular networks as sensors and drivers of common human diseases. Nature 461: 218–223. 19741703 10.1038/nature08454

[4] Geman D, D’Avignon C, Naiman DQ, Winslow RL (2004) Classifying gene expression profiles from pairwise mRNA comparisons. Statistical Applications in Genetics and Molecular Biology 3: Article19. 16646797 10.2202/1544-6115.1071 PMC1989150

[5] Tan AC, Naiman DQ, Xu L, Winslow RL, Geman D (2005) Simple decision rules for classifying human cancers from gene expression profiles. Bioinformatics 21: 3896–3904. 16105897 10.1093/bioinformatics/bti631 PMC1987374

[6] Chopra P, Lee J, Kang J, Lee S (2010) Improving cancer classification accuracy using gene pairs. PLoS ONE 5: e14305. 21200431 10.1371/journal.pone.0014305 PMC3006158

[7] Lin X, Afsari B, Marchionni L, Cope L, Parmigiani G, et al (2009) The ordering of expression among a few genes can provide simple cancer biomarkers and signal BRCA1 mutations. BMC bioinformatics 10: 256. 19695104 10.1186/1471-2105-10-256 PMC2745389

[8] Magis AT, Price ND (2012) The top-scoring ‘N’ algorithm: a generalized relative expression classification method from small numbers of biomolecules. BMC bioinformatics 13: 227. 22966958 10.1186/1471-2105-13-227 PMC3663421

[9] Wang H, Zhang H, Dai Z, Chen Ms, Yuan Z (2013) TSG: a new algorithm for binary and multi-class cancer classification and informative genes selection. BMC medical genomics 6 Suppl 1S3. 10.1186/1755-8794-6-S1-S3 PMC3552704 23445528

[10] Subramanian A, Tamayo P, Mootha VK, Mukherjee S, Ebert BL, et al (2005) Gene set enrichment analysis: A knowledge-based approach for interpreting genome-wide expression profiles. Proceedings of the National Academy of Sciences of the United States of America 102: 15545–15550. 16199517 10.1073/pnas.0506580102 PMC1239896

[11] Kanehisa M, Goto S, Sato Y, Furumichi M, Tanabe M (2012) KEGG for integration and interpretation of large-scale molecular data sets. Nucleic acids research 40: D109–14. 22080510 10.1093/nar/gkr988 PMC3245020

[12] Franceschini A, Szklarczyk D, Frankild S, Kuhn M, Simonovic M, et al (2013) STRING v9.1: protein-protein interaction networks, with increased coverage and integration. Nucleic acids research 41: D808–15. 23203871 10.1093/nar/gks1094 PMC3531103

[13] Chuang HY, Lee E, Liu YT, Lee D, Ideker T (2007) Network-based classification of breast cancer metastasis. Molecular Systems Biology 3: 140. 17940530 10.1038/msb4100180 PMC2063581

[14] Chowdhury SA, Koyutürk M (2010) Identification of coordinately dysregulated subnetworks in complex phenotypes. Pacific Symposium On Biocomputing 144: 133–144. 10.1142/9789814295291_0016 19908366

[15] Dao P, Colak R, Salari R, Moser F, Davicioni E, et al (2010) Inferring cancer subnetwork markers using density-constrained biclustering. Bioinformatics 26: i625–i631. 20823331 10.1093/bioinformatics/btq393 PMC2935415

[16] Su J, Yoon BJ, Dougherty ER (2010) Identification of diagnostic subnetwork markers for cancer in human protein-protein interaction network. BMC Bioinformatics 11: S8. 10.1186/1471-2105-11-S6-S8 PMC3026382 20946619

[17] Eddy JA, Hood L, Price ND, Geman D (2010) Identifying Tightly Regulated and Variably Expressed Networks by Differential Rank Conservation (DIRAC). PLoS Computational Biology 6: 17. 10.1371/journal.pcbi.1000792 PMC2877722 20523739

[18] Clauset A, Newman MEJ, Moore C (2004) Finding community structure in very large networks. Physical Review E 70: 1–6. 10.1103/PhysRevE.70.066111 15697438

[19] Stalteri MA, Harrison AP (2007) Interpretation of multiple probe sets mapping to the same gene in Affymetrix GeneChips. BMC Bioinformatics 8: 13. 17224057 10.1186/1471-2105-8-13 PMC1784106

[20] Golub TR, Slonim DK, Tamayo P, Huard C, Gaasenbeek M, et al (1999) Molecular classification of cancer: class discovery and class prediction by gene expression monitoring. Science 286: 531–537. 10521349 10.1126/science.286.5439.531

[21] Pomeroy SL, Tamayo P, Gaasenbeek M, Sturla LM, Angelo M, et al (2002) Prediction of central nervous system embryonal tumour outcome based on gene expression. Nature 415: 436–442. 11807556 10.1038/415436a

[22] Shipp MA, Ross KN, Tamayo P, Weng AP, Kutok JL, et al.. (2002) Diffuse large B-cell lymphoma outcome prediction by gene-expression profiling and supervised machine learning. Technical Report 1, Dana-Farber Cancer Institute, Harvard Medical School, Boston, Massachusetts, USA. margaret shipp@dfci.harvard.edu. 10.1038/nm0102-68 11786909

[23] Singh D, Febbo PG, Ross K, Jackson DG, Manola J, et al (2002) Gene expression correlates of clinical prostate cancer behavior. Cancer Cell 1: 203–209. 12086878 10.1016/s1535-6108(02)00030-2

[24] Welsh JB, Sapinoso LM, Su AI, Kern SG, Wang-rodriguez J, et al (2001) Analysis of Gene Expression Identifies Candidate Markers and Pharmacological Targets in Prostate Cancer. Cancer Research 61: 5974–5978. 11507037

[25] Gordon GJ, Jensen RV, Hsiao LL, Gullans SR, Blumenstock JE, et al (2002) Translation of microarray data into clinically relevant cancer diagnostic tests using gene expression ratios in lung cancer and mesothelioma. Cancer Research 62: 4963–4967. 12208747

[26] Ramaswamy S, Tamayo P, Rifkin R, Mukherjee S, Yeang CH, et al (2001) Multiclass cancer diagnosis using tumor gene expression signatures. Proceedings of the National Academy of Sciences of the United States of America 98: 15149–15154. 11742071 10.1073/pnas.211566398 PMC64998

[27] Ward JH (1963) Hierarchical grouping to optimize an objective function. Journal of the American Statistical Association 58: 236–244.

[28] Mardia KV, Kent JT, Bibby JM (1979) Multivariate analysis. Academic Press.

[29] Mudunuri U, Che A, Yi M, Stephens RM (2009) bioDBnet: the biological database network. Bioinformatics 25: 555–556. 19129209 10.1093/bioinformatics/btn654 PMC2642638

[30] Dos Santos C, Demur C, Bardet V, Prade-Houdellier N, Payrastre B, et al (2008) A critical role for Lyn in acute myeloid leukemia. Blood 111: 2269–79. 18056483 10.1182/blood-2007-04-082099

[31] Park S, Chapuis N, Tamburini J, Bardet V, Cornillet-Lefebvre P, et al (2010) Role of the PI3K/AKT and mTOR signaling pathways in acute myeloid leukemia. Haematologica 95: 819–28. 19951971 10.3324/haematol.2009.013797 PMC2864389

[32] Tamburini J, Chapuis N, Bardet V, Park S, Sujobert P, et al (2008) Mammalian target of rapamycin (mTOR) inhibition activates phosphatidylinositol 3-kinase/Akt by up-regulating insulinlike growth factor-1 receptor signaling in acute myeloid leukemia: rationale for therapeutic inhibition of both pathways. Blood 111: 379–82. 17878402 10.1182/blood-2007-03-080796

[33] Oellerich T, Oellerich MF, Engelke M, Münch S, Mohr S, et al (2013) *β*2 integrin-derived signals induce cell survival and proliferation of AML blasts by activating a Syk/STAT signaling axis. Blood 121: 3889–99. 23509157 10.1182/blood-2012-09-457887

[34] Sukhai MA, Prabha S, Hurren R, Rutledge AC, Lee AY, et al (2013) Lysosomal disruption preferentially targets acute myeloid leukemia cells and progenitors. The Journal of clinical investigation 123: 315–28. 23202731 10.1172/JCI64180 PMC3533286

[35] Sing T, Sander O, Beerenwinkel N, Lengauer T (2005) ROCR: visualizing classifier performance in R. Bioinformatics (Oxford, England). 21: 3940–1. 10.1093/bioinformatics/bti623 16096348

