# Supplementary material for: Automatic Context-Specific Subnetwork Discovery from Large Interaction Networks
Source: PLoS One. 2014 Jan 1;9(1):e84227. doi: 10.1371/journal.pone.0084227 (PMC3877685; doi:10.1371/journal.pone.0084227)
Supplement: Document S1 — Supporting information document. This document contains seven sections (S1. Estimated t-score, S2. Binary Vote, S3. Dataset Download Sources, S4. An Experiment with the appropriate range, S5. Algorithm Illustration, S6. Irregular Network Topology, and S7. Representative probesets of the top MISs), two figures (S1. An illustration of the MIS generation, and S2. Topology of out-of-size MISs), and five Tables (S1. Dataset Download Sources, S2. LOOCV accuracy with different appropriate ranges from KEGG, S3. LOOCV accuracy with different appropriate ranges from STRING, S4. Representative probesets of the top MISs from KEGG, and S5. Representative probesets of the top MISs from STRING). (PDF) [file pone.0084227.s001.pdf]

Automatic Context-Specific Subnetwork Discovery from Large Interaction Networks (Supporting Information)

Ashis Saha, Aik Choon Tan, Jaewoo Kang

Table of Contents

S1.Estimated t-score **2**

S2.Binary Vote **3**

S3.Dataset Download Sources **4**
Table S1. Dataset Download Sources 4

S4.An Experiment with the appropriate range **5**
Table S2. LOOCV accuracy with different appropriate ranges from KEGG 5
Table S3. LOOCV accuracy with different appropriate ranges from STRING 5

S5.Algorithm Illustration **6**
Figure S1. An illustration of the MIS generation. 6

S6.Irregular Network Topology **7**
Figure S2. Topology of out-of-size MISs. 7

S7.Representative probesets of the top MISs **8**
Table S4. Representative probesets of the top MISs from KEGG. 8
Table S5. Representative probesets of the top MISs from STRING. 12

S1. Estimated t-score

The Welch's t-test is a widely used metric for measuring the differential expression of a probe or gene (see Eq. S1).

$$t = \frac{\bar{X}_+ - \bar{X}_-}{\sqrt{\frac{{\sigma_+}^2}{n_+} + \frac{{\sigma_-}^2}{n_-}}} \tag{S1}$$

where $\bar{X}_+$ and $\bar{X}_-$ are the mean expressions, σ_+ and σ_- are the standard deviations, and n_+ and n_- are the number of samples of positive and negative classes, respectively. The higher $|t|$ is, the higher the differential power. We use a slightly modified version of the t-test to avoid the noise of the microarray data. We use the median instead of the mean, and we estimate the standard deviation from the interquartile range (IQR) which is defined as the difference between the upper and lower quantiles. The IQR contains 50% of the data within $\frac{1}{2}IQR$ of the median. Our estimation comes from the *empirical rule* - about 68.2% of the values of a normal distribution lie within 1 standard deviation of the mean. The estimated standard deviation ($\hat{\sigma}$) is given by Eq. S2,

$$\hat{\sigma} = \frac{1}{2} \cdot IQR \cdot \frac{68.2}{50} \tag{S2}$$

So, our estimated t-test score ($\hat{t}$) is given by Eq. S3.

$$\hat{t} = \frac{\tilde{X}_+ - \tilde{X}_-}{\sqrt{\frac{\hat{\sigma}_+^2}{n_+} + \frac{\hat{\sigma}_-^2}{n_-}}} \tag{S3}$$

where $\tilde{X}_+$ and $\tilde{X}_-$ are the median expressions, $\hat{\sigma}_+$ and $\hat{\sigma}_-$ are the estimated standard deviations, and n_+ and n_- are the number of samples of positive and negative classes, respectively. The higher $|\hat{t}|$ is, the higher the differential power. We sort the probes of each MIS according to the absolute value of the estimated t-scores ($|\hat{t}|$) in decreasing order, and select the top five probes as the representative probeset for the corresponding MIS.

S2. Binary Vote

Binary voting is applied when the voting weights for both classes become equal, which would be very infrequent. In the binary voting system, each top MIS casts a vote in favor of either the positive or negative class, i.e., the voting weight for each class will be either 1 or 0. Comparable to weighted voting, which was described in the main paper, binary voting for a new sample is also determined from the closest cluster. The majority class in the closest cluster gets the total vote (weight=1). If $\hat{P}_c > \hat{N}_c$, then $W_i(positive) = 1$ and $W_i(negative) = 0$. Similarly, if $\hat{P}_c < \hat{N}_c$, then $W_i(positive) = 0$ and $W_i(negative) = 1$. If $\hat{P}_c = \hat{N}_c$, the voting weight is determined in the same way, from the normalized number of positive and negative samples in the next closest cluster from x_{new}, and so on. If T, the number of voting MISs, is odd, then $W(positive) = \sum_{i=1}^{T} W_i(positive)$ and $W(negative) = \sum_{i=1}^{T} W_i(negative)$ in binary voting will never be equal. If $W(positive) > W(negative)$, the class, $binary(x_{new})$, predicted from the binary voting is *positive*; otherwise, it is *negative*.

$$binary(x_{new}) = \begin{cases} positive & \text{if } W(positive) > W(negative) \\ negative & \text{otherwise} \end{cases} \tag{S4}$$

S3. Dataset Download Sources

Table S1. Dataset Download Sources

Dataset Name	Download Source
Leukemia	http://www.broadinstitute.org/cgi-bin/cancer/publications/pub_paper.cgi?mode=view&paper_id=43
CNS	http://www.broadinstitute.org/mpr/CNS/
DLBCL	http://www.broadinstitute.org/mpr/lymphoma/
Prostate1	http://www.broadinstitute.org/cgi-bin/cancer/publications/pub_paper.cgi?mode=view&paper_id=75
Prostate3	http://www.gnf.org/cancer/prostate (We found the original link broken on 15th November 2013. However, an alternative source is available - http://datam.i2r.a-star.edu.sg/datasets/krbd/ProstateCancer/ProstateCancer.html.)
Lung	http://datam.i2r.a-star.edu.sg/datasets/krbd/LungCancer/LungCancer-Harvard2.html
GCM	http://www.broadinstitute.org/cgi-bin/cancer/publications/pub_paper.cgi?mode=view&paper_id=61

S4. An Experiment with the appropriate range

We set the appropriate range, [$minRange, maxRange$], to generate the molecular interaction subnetworks. We experimented with different ranges and chose the optimal range producing the highest LOOCV accuracy over the datasets. Initially, we set $minRange = 3, 5, 7$ and $maxRange = 15, 20, 25$ for KEGG, and $minRange = 5, 7, 10$ and $maxRange = 15, 20, 25$ for STRING. Later, we expanded the range list based on the results observed. The results of the MISs with different appropriate ranges using KEGG and STRING are shown in Table S2 and S3, respectively.

Table S2. LOOCV accuracy (%) with different appropriate ranges from KEGG*

Appro. Range	Leukemia	CNS	DLBCL	Prostate1	Prostate3	Lung	GCM	Average
3-15	98.6	82.4	93.5	89.2	100.0	99.5	85.4	92.7
3-20	98.6	73.5	94.8	88.2	100.0	99.5	83.6	91.2
3-25	97.2	82.4	94.8	90.2	100.0	99.5	83.9	92.6
5-10	97.2	82.4	94.8	91.2	100.0	99.5	84.6	92.8
**5-15**	**98.6**	**85.3**	**93.5**	**90.2**	**100.0**	**99.5**	**85.0**	**93.2**
5-20	98.6	70.6	94.8	88.2	100.0	99.5	83.6	90.8
5-25	97.2	82.4	94.8	90.2	100.0	99.5	83.9	92.6
5-30	98.6	82.4	94.8	89.2	100.0	99.5	83.9	92.6
7-15	98.6	76.5	96.1	88.2	100.0	99.5	84.6	91.9
7-20	97.2	82.4	94.8	87.3	100.0	99.5	83.6	92.1
7-25	97.2	85.3	94.8	88.2	100.0	99.5	83.6	92.7

'Appro. Range' denotes *appropriate range*. * The optimal appropriate range producing the highest average LOOCV accuracy is shown in bold font.

Table S3. LOOCV accuracy (%) with different appropriate ranges from STRING*

Appro. Range	Leukemia	CNS	DLBCL	Prostate1	Prostate3	Lung	GCM	Average
5-10	97.2	82.4	94.8	91.2	100.0	81.4	98.9	92.3
5-15	94.4	85.3	93.5	88.2	100.0	83.6	98.9	92.0
5-20	97.2	76.5	89.6	91.2	100.0	82.9	99.5	91.0
**5-25**	**95.8**	**88.2**	**94.8**	**90.2**	**97.0**	**84.6**	**98.3**	**92.7**
5-30	97.2	88.2	88.3	91.2	100.0	84.3	98.9	92.6
7-15	98.6	76.5	94.8	88.2	97.0	98.3	83.6	91.0
7-20	98.6	76.5	92.2	89.2	100.0	98.9	83.9	91.3
7-25	94.4	82.4	88.3	89.2	97.0	98.3	84.6	90.6
7-30	95.8	82.4	92.2	91.2	100.0	99.5	84.3	92.2
10-20	95.8	79.4	93.5	89.2	100.0	98.9	83.6	91.5
10-25	97.2	79.4	88.3	91.2	100.0	98.3	82.9	91.0
10-30	97.2	79.4	94.8	91.2	100.0	98.9	83.9	92.2

'Appro. Range' denotes *appropriate range*. * The optimal appropriate range producing the highest average LOOCV accuracy is shown in bold font.

S5. Algorithm Illustration

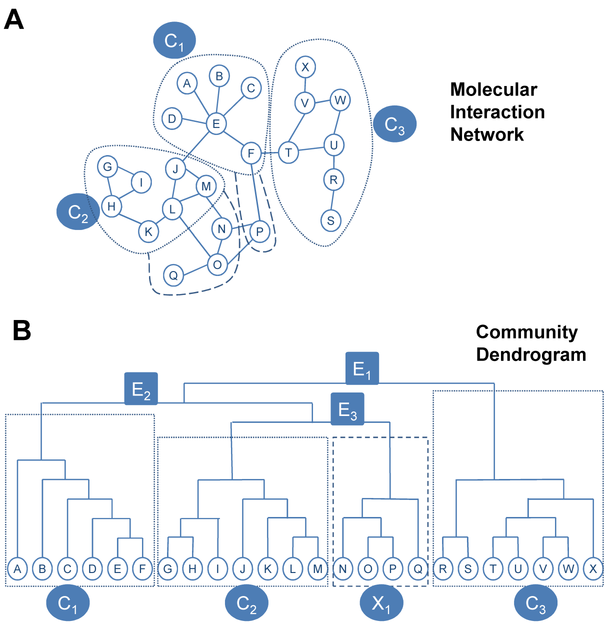

Figure S1. An illustration of the MIS generation. Let us consider one example of a connected molecular interaction network (MIS) and its community dendrogram as shown in the figure. Let the appropriate range be [5,10]. The size (the total number of leaf nodes) of the dendrogram is 24. As it is greater than the *maxRange* (10), we divide the dendrogram by removing edge E1 so we are left with two dendrograms (A-Q and R-X). The right dendrogram's (R-X) size is 7 ($5 \leq 7 \leq 10$), so we take it as an appropriate community (C_3). However, because the left dendrogram's (A-Q) size is above 10, we divide it again by removing edge E_2. We have to further divide the dendrogram by removing edge E_3. Thus we get four parts of the original community dendrogram – C_1, C_2, X_1, and C_3. Three of their sizes fall within the appropriate range [5,10] (C_1, C_2, and C_3), so we take them as appropriate communities. However, because X_1's size is less than 5, we discard it. Now, we shall assign the nodes in X_1 – N, O, P, and Q – individually to their closest communities from the original network. P is 1-hop away from C_1, so P is merged with C_1; N and O are 1-hop away from C_2, so they are merged with C_2. In the next iteration, Q is merged with its closest community, C_2. Thus, we get three MISs – C_1 (A,B,C,D,E,F,P), C_2 (G,H,I,J,K,L,M,N,O,Q), and C_3 (R,S,T,U,V,W,X).

S6. Irregular Network Topology

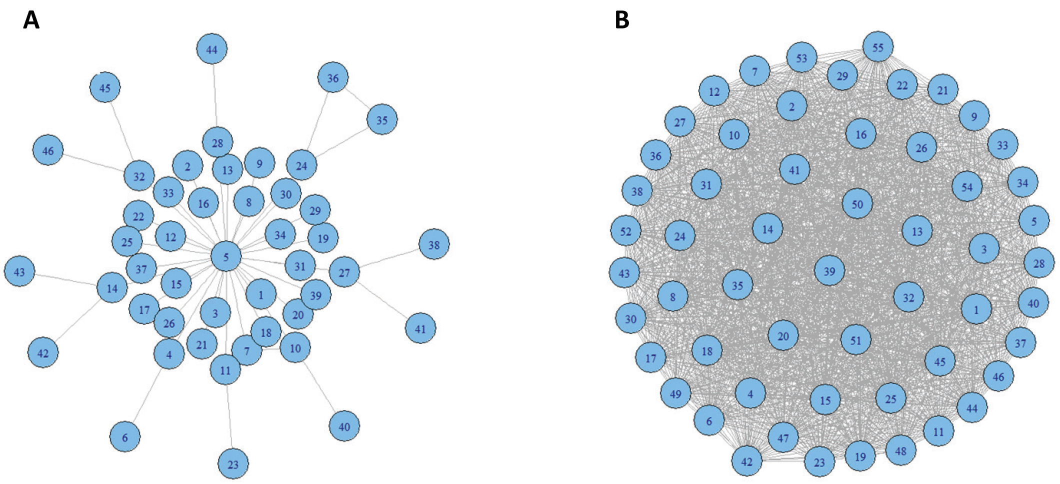

Figure S2. Topology of two out-of-size MISs generated with an appropriate range of 5-15 from STRING network. A) MIS with 46 nodes has a star topology. B) MIS with 55 nodes is too dense.

S7. Representative probesets of the top MISs

Table S4. Representative probesets of the top MISs from KEGG. MISs generated from KEGG have been ranked by COSSY using all the samples in a dataset, and the representative probesets of the top T MISs have been reported where T is the number of MISs to vote that produced the highest LOOCV accuracy. Each probeset's ID is followed by the corresponding gene symbol in parentheses.

Dataset	T	Rank	Representative probeset of MIS [probeid(gene symbol)]
Leukemia	15	1	D87459_at(WASF1), M19283_at(ACTG1), L33075_at(IQGAP1), X74008_at(PPP1CC), X95735_at(ZYX)
		2	X64072_s_at(ITGB2), M15395_at(ITGB2), M81695_s_at(ITGAX), X80907_at(PIK3R2), X51521_at(EZR)
		3	D16469_at(ATP6AP1), L09717_at(LAMP2), M63138_at(CTSD), S46622_at(PPP3CC), Z46973_at(PIK3C3)
		4	M16038_at(LYN), U14588_at(PXN), X80907_at(PIK3R2), X04526_at(GNB1), X77748_at(GRM3)
		5	U46499_at(MGST1), U77604_at(MGST2), Y00433_at(GPX1), U21689_at(GSTP1), M81118_at(ADH5)
		6	M22898_at(TP53), U22376_cds2_s_at(MYB), M65214_s_at(TCF3), M38449_s_at(TGFB1), M31523_at(TCF3)
		7	U14588_at(PXN), M24283_at(ICAM1), X06318_at(PRKCB), S72869_at(CCDC6), X77548_at(NCOA4)
		8	M16038_at(LYN), M64595_at(RAC2), M89957_at(CD79B), U05259_rna1_at(CD79A), X16316_at(VAV1)
		9	U29656_at(NME3), U52427_rna1_at(POLR2G), X15357_at(NPR1), X66533_at(GUCY1B3), Y07604_at(NME4)
		10	M63167_at(AKT1), X80907_at(PIK3R2), M18255_cds2_s_at(PRKCB), M84371_rna1_s_at(CD19), X06318_at(PRKCB)
		11	D10495_at(PRKCD), M14676_at(FYN), M16038_at(LYN), M33195_at(FCER1G), X06318_at(PRKCB)
		12	L09708_at(C2), M84526_at(CFD), X05309_at(CR1), M13690_s_at(SERPING1), U28488_s_at(C3AR1)
		13	M27281_at(VEGFA), U09578_at(MAPKAPK3), U14588_at(PXN), X80907_at(PIK3R2), Z29090_at(PIK3CA)
		14	L05147_at(DUSP3), M22898_at(TP53), X66867_cds1_at(MAX), L08895_at(MEF2C), D11327_s_at(PTPN7)
		15	M95678_at(PLCB2), U03090_at(PLA2G5), X04828_at(GNAI2), U38545_at(PLD1), X51521_at(EZR)
CNS	7	1	J04177_at(COL11A1), M55210_at(LAMC1), Z26653_at(LAMA2), Z74616_s_at(COL1A2), X79683_s_at(LAMB2)
		2	M81933_at(CDC25A), U31814_at(HDAC2), U18422_at(TFDP2), L40386_s_at(TFDP2), S49592_s_at(E2F1)
		3	M55210_at(LAMC1), X79683_s_at(LAMB2), X07979_at(ITGB1), U04806_s_at(FLT3LG), L32137_at(COMP)
		4	J04111_at(JUN), M92287_at(CCND3), L00058_at(MYC), M25269_at(ELK1), M14745_at(BCL2)
		5	L36033_at(CXCL12), U09303_at(EFNB1), U27655_at(RGS3), U81262_at(EFNB2), L06797_s_at(CXCR4)

		6	D25538_at(ADCY7), L21993_at(ADCY2), M82919_at(GABRB3), X15376_at(GABRG2), U73304_rna1_at(CNR1)
		7	J04177_at(COL11A1), M60299_at(COL2A1), Z74616_s_at(COL1A2), L32137_at(COMP), X07979_at(ITGB1)
DLBCL	7	1	S74728_at(ALDH7A1), U40369_rna1_at(SAT1), X05409_at(ALDH2), Z49099_at(SMS), M34338_s_at(SRM)
		2	X13444_at(LOC100996919), X56841_at(HLA-E), D49824_s_at(HLA-B), M94880_f_at(HLA-H), X57522_at(TAP1)
		3	D21063_at(MCM2), D55716_at(MCM7), D84557_at(MCM6), U77949_at(CDC6), X74795_at(MCM5)
		4	L33801_at(GSK3B), M80335_at(PRKACA), U37352_at(PPP2R5C), X12791_at(APC), M60483_rna1_s_at(PPP2CA)
		5	U21090_at(POLD2), X17620_at(NME1), X56494_at(PKM), X59543_at(RRM1), M80397_s_at(POLD1)
		6	D31797_at(CD40LG), L33801_at(GSK3B), L41067_at(NFATC3), M37815_cds1_at(CD28), M95936_s_at(AKT2)
		7	D90084_at(PDHA1), J04173_at(PGAM1), X02152_at(LDHA), X56494_at(PKM), M14328_s_at(ENO1)
Prostate1	29	1	37599_at(AOX1), 40071_at(CYP1B1), 41772_at(MAOA), 32747_at(ALDH2), 859_at(CYP1B1)
		2	34915_at(SLC8A1), 40435_at(SLC25A6), 40436_g_at(SLC25A6), 39123_s_at(TRPC1), 39124_r_at(TRPC1)
		3	39775_at(SERPING1), 40766_at(C4A), 32249_at(CFHR1), 38441_s_at(CD46), 40282_s_at(CFD)
		4	41867_at(CREB3L1), 33134_at(ADCY3), 1909_at(BCL2), 41288_at(CALM3), 364_s_at(PLCB3)
		5	39939_at(COL4A6), 32667_at(COL4A5), 41755_at(COBLL1), 37912_at(TRAF4), 38042_at(PRKCA)
		6	41867_at(CREB3L1), 37733_at(MAPK14), 38042_at(PRKCA), 41288_at(CALM3), 1708_at(MAPK10)
		7	31824_at(ME1), 35216_at(ME3), 33819_at(LDHB), 41485_at(LDHA), 837_s_at(ME1)
		8	32747_at(ALDH2), 37707_i_at(ADH5), 37708_r_at(ADH5), 36686_at(ALDH1A3), 38780_at(AKR1A1)
		9	37186_s_at(ABP1), 41772_at(MAOA), 32747_at(ALDH2), 36132_at(ALDH7A1), 36686_at(ALDH1A3)
		10	33710_at(LPCAT3), 38262_at(MBOAT2), 39396_at(LYPLA1), 34797_at(PPAP2A), 38098_at(LPIN1)
		11	40162_s_at(COMP), 40163_r_at(COMP), 103_at(THBS4), 1767_s_at(TGFB3), 34342_s_at(SPP1)
		12	39054_at(GSTM4), 33396_at(GSTP1), 1120_at(GSTM3), 820_at(MGST2), 556_s_at(GSTM4)
		13	33994_g_at(MYL6), 32755_at(ACTA2), 41288_at(CALM3), 38298_at(KCNMB1), 33134_at(ADCY3)
		14	34138_at(GNAO1), 38042_at(PRKCA), 41288_at(CALM3), 1336_s_at(PRKCB), 36918_at(GUCY1A3)
		15	34637_f_at(ADH1A), 38315_at(ALDH1A2), 40782_at(DHRS3), 37707_i_at(ADH5), 37708_r_at(ADH5)
		16	40682_at(GYS2), 37221_at(PRKAR2B), 39366_at(PPP1R3C), 37725_at(PPP1CC), 41288_at(CALM3)

		17	AFFX-HUMGAPDH/M33197_M_at(GAPDH), 33819_at(LDHB), 40193_at(ENO2), 41485_at(LDHA), 2035_s_at(ENO1)
		18	36917_at(LAMA2), 36929_at(LAMB3), 41755_at(COBLL1), 33410_at(ITGA6), 37671_at(LAMA4)
		19	34118_at(ATP1B2), 32225_at(ATP1A1), 34377_at(ATP1A2), 39356_at(NEDD4L), 34915_at(SLC8A1)
		20	1767_s_at(TGFB3), 1735_g_at(TGFB3), 1262_s_at(TGFB2), 41140_at(IFNGR2), 1038_s_at(IFNGR1)
		21	1767_s_at(TGFB3), 1622_at(MAP2K3), 857_at(PPM1A), 37408_at(MRC2), 806_at(PLK3)
		22	36686_at(ALDH1A3), 37708_r_at(ADH5), 1120_at(GSTM3), 556_s_at(GSTM4), 40071_at(CYP1B1)
		23	41385_at(EPB41L3), 38042_at(PRKCA), 1336_s_at(PRKCB), 208_at(CTNNA2), 36174_at(MARCKSL1)
		24	35649_at(CDO1), 33819_at(LDHB), 36123_at(TST), 36124_at(MPST), 41485_at(LDHA)
		25	40435_at(SLC25A6), 40436_g_at(SLC25A6), 35818_at(CYCS), 486_at(CASP9), 32806_at(TSPO)
		26	41422_at(DPYS), 37193_at(MIR3658), 37351_at(UPP1), 33815_at(UMPS), 37203_at(CES1)
		27	34145_at(TLR6), 36243_at(TLR1), 40310_at(TLR2), 37720_at(HSPD1), 41510_s_at(HSPA9)
		28	37229_at(ATR), 36645_at(RELA), 1767_s_at(TGFB3), 1295_at(RELA), 1100_at(IRAK1)
		29	1661_i_at(KLK3), 1513_at(KLK3), 1514_g_at(KLK3), 442_at(MIR3652), 1664_at(IGF2)
Prostate3	3	1	32962_at(CTH), 33819_at(LDHB), 35343_at(GOT1), 36124_at(MPST), 41485_at(LDHA)
		2	40372_at(PNLIPRP1), 38003_s_at(DGKZ), 39044_s_at(DGKD), 33862_at(PPAP2B), 38098_at(LPIN1)
		3	35118_at(LCAT), 33710_at(LPCAT3), 35228_at(CHKB), 39396_at(LYPLA1), 41176_at(LPCAT1)
Lung	7	1	34664_at(FCGR2B), 35822_at(CFB), 39409_at(C1R), 40496_at(C1S), 40766_at(C4A)
		2	33904_at(CLDN3), 35276_at(CLDN4), 35630_at(LLGL2), 38482_at(CLDN7), 40973_at(PARD3)
		3	35330_at(FLNC), 36354_at(TNN), 36917_at(LAMA2), 36929_at(LAMB3), 881_at(ITGB6)
		4	1810_s_at(PRKCD), 32046_at(PRKCD), 33833_at(SPTAN1), 767_at(MYH11), 774_g_at(MYH11)
		5	1810_s_at(PRKCD), 32046_at(PRKCD), 41645_at(PLA2G6), 33245_at(MAPK13), 33804_at(PTK2B)
		6	1108_s_at(EPHA1), 2039_s_at(FYN), 34329_at(PAK2), 39930_at(EPHB6), 40425_at(EFNA1)
		7	1810_s_at(PRKCD), 32046_at(PRKCD), 33162_at(INSR), 41049_at(IRS1), 851_s_at(IRS1)
GCM	23	1	X04828_at(GNAI2), X70297_at(CHRNA7), Y10141_s_at(SLC6A3), AA278775_at(APLP2), RC_AA459690_s_at(MPC1)

2	M58603_at(NFKB1), U33053_at(PKN1), U70451_at(MYD88), S75881_s_at(MYBL1), H39589_at(COQ4)
3	L76191_at(IRAK1), M11717_rna1_at(HSPA1B), X51757_at(HSPA6), AA489287_at(FBXO16), RC_AA398708_at(SEPT7)
4	L11285_at(MAP2K2), M21574_at(PDGFRA), X02751_at(NRAS), X04828_at(GNAI2), RC_AA147646_s_at(METTL7A)
5	D00632_at(GPX3), Y00433_at(GPX1), L16991_at-2(CYP2C9), M30185_at-2(CYP2B6), U78310_at(PES1)
6	D43767_at(CCL17), U85767_at(CCL23), X04828_at(GNAI2), X55989_rna1_at(ECRP), U75285_rna1_at(BIRC5)
7	M21056_at(PLA2G1B), M34667_at(PLCG1), U61538_at(CHP1), U79271_at(AKT3), RC_AA609519_at(MSRA)
8	L11005_at(AOX1), U89606_at(PDXK), RC_AA400074_at(DOCK8), RC_AA426643_at(RSL1D1), RC_AA428608_at(ROGDI)
9	M22995_at(RAP1A), M34667_at(PLCG1), Z15114_at(PRKCG), M24485_s_at(GSTP1), W69543_at(SCML1)
10	L01087_at(PRKCQ), L11285_at(MAP2K2), M34353_s_at(ROS1), M34667_at(PLCG1), RC_AA029462_at(RALGAPA2)
11	X55733_at(EIF4B), M33666_at(PSG6), M61733_at-2(RPS6KB1), RC_AA400766_at(KIAA0556), RC_AA416601_s_at(ULK3)
12	X81817_at(BCAP31), Z12830_at(SSR1), AA093396_at(PHGDH), T69384_at(PER1), RC_AA443342_s_at(TMEM203)
13	M21186_at(CYBA), M55067_at(NCF1B), X77094_at(NCF4), M30448_s_at(FBL), U05875_at(IFNGR2)
14	M16038_at(LYN), M34667_at(PLCG1), U20158_at(LCP2), U78027_rna4_at(BTK), X06948_at(FCER1A)
15	L76191_at(IRAK1), U91616_at(NFKBIE), X69550_at(ARHGDIA), HG3996-HT4266_at(ARHGDIB), RC_AA261907_at(REXO2)
16	M11717_rna1_at(HSPA1B), X51757_at(HSPA6), AA465601_at(ARFIP1), X15875_at(ATF2), AA422159_at(DOHH)
17	D31766_at(GNPDA1), RC_AA035284_at(PDZRN3), RC_AA210695_at(PARP14), RC_AA429655_at(TPCN1), RC_AA449479_at(BZW2)
18	U10473_s_at(B4GALT1), U28014_at(CASP4), RC_AA429655_at(TPCN1), RC_AA443841_at(SPRY2), R93273_s_at(UXS1)
19	X16663_at(HCLS1), X01677_f_at(GAPDH), V00565_s_at-2(ACTB), RC_AA402656_at(ZCCHC24), RC_AA609519_at(MSRA)
20	U67733_at(PDE2A), Y00486_rna1_at(APRT), AA318315_at(C6orf106), RC_AA609873_at(NECAB3), M91029_cds2_at(AMPD2)
21	L11285_at(MAP2K2), M22995_at(RAP1A), M95712_at(BRAF), X02751_at(NRAS), AA278775_at(APLP2)
22	M21056_at(PLA2G1B), X04828_at(GNAI2), F07806_at(GNAZ), RC_AA193204_at(SORBS2), M16038_at(LYN)
23	M95740_at(IDUA), U03735_f_at(MAGEA6), RC_AA193204_at(SORBS2), L33798_at(CACNA1S), RC_AA459690_s_at(MPC1)

Table S5. Representative probesets of the top MISs from STRING. MISs generated from STRING have been ranked by COSSY using all the samples in a dataset, and the representative probesets of the top T MISs have been reported where T is the number of MISs to vote that produced the highest LOOCV accuracy. Each probeset's ID is followed by the corresponding gene symbol in parentheses.

Dataset	T	Rank	Representative probeset of MIS [probeid(gene symbol)]
Leukemia	5	1	L21954_at(TSPO), M54992_at(CD72), U05259_rna1_at(CD79A), X07743_at(PLEK), X97267_rna1_s_at(PTPRCAP)
		2	J03473_at(PARP1), U25435_at(CTCF), X99585_at(SUMO2), Z15115_at(TOP2B), M27504_s_at(TOP2B)
		3	X92106_at(BLMH), Z23064_at(SNORD61), Z93784_at(ATXN10), M21535_at(ERG), L09209_s_at(APLP2)
		4	L09717_at(LAMP2), M22960_at(CTSA), M74524_at(UBE2A), U76992_at(HTATSF1), S79873_s_at(LAMP2)
		5	D87465_at(SPOCK2), D83646_at(MMP16), U14394_at(TIMP3), D50477_s_at(MMP16), M32304_s_at(TIMP2)
CNS	5	1	L27560_at(IGFBP5), L42450_at(PDK1), M14745_at(BCL2), M65062_at(IGFBP5), M62782_s_at(IGFBP5)
		2	X04143_at(BGLAP), Z74615_at(COL1A1), D13666_s_at(POSTN), Z74616_s_at(COL1A2), M55998_s_at(COL1A1)
		3	D14838_at(FGF9), L40027_at(GSK3A), M93650_at(PAX6), U07223_at(CHN2), X14474_at(MAPT)
		4	L06139_at(TEK), M55593_at(MMP2), U89336_cds3_at(AGER), X57766_at(MMP11), U31903_s_at(ATF6B)
		5	M19720_rna2_at(MYCL1), M95929_at(SFXN3), U22377_at(RLF), L00058_at(MYC), L07648_at(MXI1)
DLBCL	17	1	D78134_at(CIRBP), U23803_at(HNRNPA0), M60784_s_at(SNRPA), X15729_s_at(DDX5), X74874_rna1_s_at(POLR2A)
		2	J04615_at(SNURF), U08377_at(SFSWAP), U81001_at(SNURF), Z69915_at(RBMXL1), X17567_s_at(SNRPB)
		3	D21063_at(MCM2), D80008_at(GINS1), U77949_at(CDC6), U37426_at(KIF11), X85137_s_at(KIF11)
		4	J03909_at(IFI30), U15590_at(HSPB3), X03934_at(CD3D), X04145_at(CD3G), M23323_s_at(CD3E)
		5	D38553_at(NCAPH), D79997_at(MELK), M74558_at(STIL), X51688_at(CCNA2), X67155_at(KIF23)
		6	D87292_at(TST), X02152_at(LDHA), X05409_at(ALDH2), Z22548_at(PRDX2), M34338_s_at(SRM)
		7	J04988_at(HSP90AB1), U12595_at(TRAP1), U24169_at(AIMP2), U41387_at(DDX21), X75861_at(TMBIM6)
		8	D31890_at(KARS), M63180_at(TARS), U07424_at(FARSA), U09510_s_at(GARS), U09587_at(GARS)
		9	U80040_at(ACO2), V00572_at(PGK1), X07834_at(SOD2), Z68129_cds1_at(IDH3G), X65965_s_at(SOD2)
		10	AB003177_at(PSMD9), D26599_at(PSMB2), D38047_at(PSMD8), D78151_at(PSMD2), X71874_cds1_at(PSMB10)
		11	K02268_at(PDYN), L42324_at(GPR18), U94320_at(NPY5R), L07615_at(NPY1R), U28488_s_at(C3AR1)

		12	D64142_at(H1FX), D83243_at(NPAT), L29008_at(SORD), M68520_at(CDK2), X03473_at(H1F0)
		13	M33195_at(FCER1G), M37033_at(CD53), M60830_at(EVI2B), X62466_at(CD52), U19557_s_at(SERPINB4)
		14	D90084_at(PDHA1), D90086_at(PDHB), U46692_rna1_at(CSTB), X56494_at(PKM), L12760_s_at(PCK1)
		15	D21262_at(NOLC1), D25218_at(RRS1), J03798_at(SNRPD1), U10323_at(ILF2), X70683_at(SOX4)
		16	D00596_at(TYMS), D25547_at(PCMT1), L16991_at(DTYMK), X53793_at(PAICS), X59543_at(RRM1)
		17	D14811_at(MAD2L1BP), M86699_at(TTK), U65410_at(MAD2L1), U96131_at(TRIP13), L40384_s_at(TRIP13)
Prostate1	17	1	39640_at(GFPT2), 39729_at(PRDX2), 32747_at(ALDH2), 37720_at(HSPD1), 41485_at(LDHA)
		2	34602_at(FCN2), 37085_g_at(LYZL6), 39590_at(APBA2), 40778_at(HSD17B10), 35277_at(SPON1)
		3	41115_s_at(BAIAP3), 36943_r_at(PLAGL1), 38406_f_at(PTGDS), 828_at(PTGER2), 216_at(PTGDS)
		4	39939_at(COL4A6), 32667_at(COL4A5), 37890_at(CD47), 34391_at(IGBP1), 753_at(NID2)
		5	35497_at(TSPAN2), 35631_at(POLR2H), 33415_at(NME2), 1980_s_at(NME2), 1248_at(POLR2H)
		6	39701_at(PEG3), 39755_at(XBP1), 39756_g_at(XBP1), 40856_at(SERPINF1), 38108_at(EGFL8)
		7	39353_at(HSPE1), 33904_at(CLDN3), 34348_at(SPINT2), 1513_at(KLK3), 575_s_at(EPCAM)
		8	41627_at(SDF2), 32051_at(ALG8), 36897_at(MLC1), 38057_at(DPT), 38059_g_at(DPT)
		9	39054_at(GSTM4), 1289_at(GSTM5), 1120_at(GSTM3), 820_at(MGST2), 556_s_at(GSTM4)
		10	38950_r_at(MMP23B), 35306_at(DHX15), 35766_at(KRT18), 41250_at(AIMP2), 1199_at(SNORA48)
		11	33328_at(HEG1), 35218_at(PDCD10), 35695_at(LYST), 35696_s_at(LYST), 38634_at(RBP1)
		12	32288_r_at(KLRC3), 32297_s_at(KLRC1), 40024_at(STAC), 40775_at(ITM2A), 41237_at(HLA-A)
		13	36252_at(CTF1), 39315_at(ANGPT1), 32640_at(ICAM1), 36939_at(GPM6A), 1929_at(ANGPT1)
		14	35071_s_at(GMDS), 35214_at(UGDH), 36515_at(GNE), 36936_at(TSTA3), 41242_at(UAP1)
		15	36780_at(CLU), 36690_at(NR3C1), 37658_at(GAS6), 1598_g_at(GAS6), 1389_at(MME)
		16	34608_at(SNORD95), 34609_g_at(SNORD95), 37343_at(ITPR3), 39123_s_at(TRPC1), 39124_r_at(TRPC1)
		17	34735_at(ROCK1), 37276_at(IQGAP2), 38292_at(HOMER2), 40512_at(CHN1), 1647_at(IQGAP2)
Prostate3	5	1	41640_at(YIPF1), 34870_at(LDB3), 38422_s_at(FHL2), 38736_at(WDR1), 32542_at(FHL1)
		2	37832_at(MMACHC), 41191_at(PALLD), 41195_at(LPP), 1832_at(MCC), 794_at(PTPN6)

		3	41626_at(TIMELESS), 37646_at(POLD3), 35326_at(YIF1A), 1470_at(POLD2), 860_at(MSH2)
		4	37809_at(HOXA9), 41388_at(MEIS2), 32063_at(PBX1), 40763_at(MEIS1), 33355_at(PBX1)
		5	40118_at(ZNF3), 33454_at(AGRN), 34377_at(ATP1A2), 37669_s_at(ATP1B1), 39526_at(PAIP2B)
Lung	5	1	1500_at(WT1), 34218_at(SPEG), 376_at(SEMA3C), 377_g_at(SEMA3C), 977_s_at(CDH1)
		2	1661_i_at(KLK3), 1662_r_at(KLK3), 33904_at(CLDN3), 38482_at(CLDN7), 575_s_at(EPCAM)
		3	2056_at(FGFR1), 2057_g_at(FGFR1), 36522_at(CRTC1), 39750_at(PARVA), 39930_at(EPHB6)
		4	1237_at(IER3), 33243_at(TNFAIP8), 33900_at(FSTL3), 35694_at(MAP4K4), 37716_at(CD200)
		5	1564_at(AKT1), 1616_at(FGF9), 32260_at(PEA15), 36095_at(CLIP3), 36418_at(PRICKLE3)
GCM	11	1	D84110_at(RBPMS), L40393_at(NUMB), R78309_at(ATXN1L), RC_AA280004_at(QKI), RC_AA291162_at(SPEN)
		2	X14850_at(H2AFX), Y10262_s_at(EYA3), AA252929_at(H2AFX), AA418662_at(RINT1), RC_AA599683_at(EYA3)
		3	X67337_at(CPSF6), X76770_at(PAPOLA), AA259021_s_at(MLLT6), RC_AA179826_at(TSEN15), RC_AA463934_at(SF3B4)
		4	L17131_rna1_at(HMGA1), U47054_at(ART3), AA044715_at(ZFR), RC_AA256263_at(HNRNPH3), RC_AA449974_at(SEPT4)
		5	AB000816_s_at(ARNTL), T69384_at(PER1), W69582_at(FBXW11), RC_AA481037_at(PER3), RC_AA488844_f_at(FBXL3)
		6	L34219_at(RLBP1), M35128_at(CHRM1), M21142_cds2_s_at(GNAS), AF007216_at(SLC4A4), RC_AA452263_at(GNA11)
		7	U33053_at(PKN1), Z80787_at(HIST4H4), AA018847_at(NLK), AA287291_at(IWS1), RC_AA194257_f_at(BRD1)
		8	Z97054_xpt2_at(HUWE1), AA278775_at(APLP2), AA477046_at(UBA6), RC_AA069456_at(PJA2), RC_AA402613_at(PELI3)
		9	U22055_at(SND1), AA028976_at(DLC1), RC_AA233257_at(TGFB1I1), RC_AA487576_at(SDPR), RC_AA496366_at(TNS1)
		10	D78134_at(CIRBP), L38696_at(RALY), U03891_at(APOBEC3B), X92715_at(ZNF74), M60784_s_at(SNRPA)
		11	U08815_at(SF3A3), U15782_at(CSTF3), X13482_at(SNRPA1), X16135_at(HNRNPL), H58970_at(CPSF2)
